# Myofibroblasts and the extracellular matrix network in post-myocardial infarction cardiac remodeling

**DOI:** 10.1007/s00424-014-1463-9

**Published:** 2014-02-13

**Authors:** Yonggang Ma, Lisandra E. de Castro Brás, Hiroe Toba, Rugmani Padmanabhan Iyer, Michael E. Hall, Michael D. Winniford, Richard A. Lange, Suresh C. Tyagi, Merry L. Lindsey

**Affiliations:** 1 San Antonio Cardiovascular Proteomics Center, San Antonio, TX USA; 2 Mississippi Center for Heart Research, Department of Physiology and Biophysics, University of Mississippi Medical Center, Jackson, MS USA; 3 Cardiology Division, University of Mississippi Medical Center, Jackson, MS USA; 4 Department of Medicine, University of Texas Health Science Center at San Antonio, San Antonio, TX USA; 5 Department of Physiology and Biophysics, University of Louisville, Louisville, KY USA; 6 Research and Medicine Services, G.V. (Sonny) Montgomery Veterans Affairs Medical Center, Jackson, MS USA; 7 Department of Physiology and Biophysics, University of Mississippi Medical Center, 2500 North State St., Jackson, MS 39216-4505 USA; 8 Department of Clinical Pharmacology, Division of Pathological Sciences, Kyoto Pharmaceutical University, Kyoto, Japan

**Keywords:** Extracellular matrix, MMP-9, Myocardial infarction, Myofibroblast, Proteomics, Review

## Abstract

The cardiac extracellular matrix (ECM) fills the space between cells, supports tissue organization, and transduces mechanical, chemical, and biological signals to regulate homeostasis of the left ventricle (LV). Following myocardial infarction (MI), a multitude of ECM proteins are synthesized to replace myocyte loss and form a reparative scar. Activated fibroblasts (myofibroblasts) are the primary source of ECM proteins, thus playing a key role in cardiac repair. A balanced turnover of ECM through regulation of synthesis by myofibroblasts and degradation by matrix metalloproteinases (MMPs) is critical for proper scar formation. In this review, we summarize the current literature on the roles of myofibroblasts, MMPs, and ECM proteins in MI-induced LV remodeling. In addition, we discuss future research directions that are needed to further elucidate the molecular mechanisms of ECM actions to optimize cardiac repair.

## Introduction

Cardiovascular disease is an increasing burden worldwide, and myocardial infarction (MI) contributes significantly to morbidity and mortality. In-hospital survival for MI patients has greatly improved over the past three decades due to the introduction of reperfusion and other therapeutic strategies. However, as short-term survival after acute MI has improved, the incidence of heart failure post-MI has risen [34]. In response to MI, the left ventricle (LV) undergoes a series of changes in geometry and function, a process termed LV remodeling. Understanding the mechanisms of adverse LV remodeling is critical to our effort to improve long-term outcome after MI.

After 30 min of ischemia, cardiomyocytes undergo irreversible cell death, which induces an acute inflammatory response through upregulation of the complement pathway. Neutrophils and macrophages sequentially infiltrate the infarct region and release inflammatory mediators and matrix metalloproteinases (MMPs) to degrade extracellular matrix (ECM) and engulf tissue debris [70]. The LV has limited regenerative capacity, and a collagen-rich reparative scar is formed to replace the extensive loss of cardiomyocytes in the infarct zone [68]. Myofibroblasts are the major source of ECM in the scar. In response to inflammatory mediators, resident cardiac fibroblasts and other cell lineages are stimulated to differentiate into myofibroblasts, which results in abundant ECM synthesis and deposition [106]. The proper balance between ECM synthesis and degradation is critical for optimal infarct healing. Endogenous tissue inhibitors of metalloproteinases (TIMPs) block excessive MMP activity and play a key role in maintaining the balance between ECM synthesis and degradation. Excessive ECM accumulation increases wall stiffness and impairs compliance, leading to diastolic dysfunction, whereas inadequate ECM deposition and impaired collagen assembly facilitates progressive infarct wall thinning and infarct expansion that can lead to LV dilation, aneurysm formation, and rupture [68, 69].

ECM is composed of structural and nonstructural matricellular proteins, as well as a proteolytic system of MMPs and TIMPs [68]. After MI, an elevation in the production of structural ECM proteins generates an infarct scar, and matricellular proteins help to organize the ECM fibrillar structure as well as regulate the inflammatory and angiogenic responses. In this review, we summarize the current knowledge on the functions of myofibroblasts, MMPs, TIMPs, and specific ECM components in the MI-induced LV remodeling, as well as discuss future research directions to better understand these processes. Dissecting the mechanisms whereby fibroblasts and ECM molecules coordinate the post-MI remodeling response may identify potential interventions to stimulate an optimal infarct healing response.

## Cardiac fibroblasts and myofibroblasts

Cardiac fibroblasts are the most numerous cell types in the heart, accounting for up to 70 % of total cell numbers in the normal LV [91]. However, the role of fibroblasts has often been overlooked in favor of the more studied cardiomyocyte. Recent studies have focused on the complex and dynamic interaction between cardiac fibroblasts, cardiomyocytes, and leukocytes in the development of cardiovascular disease [8]. Under physiological conditions, cardiac fibroblasts provide a mechanical scaffold and coordinate systolic heart function. Additionally, myofibroblasts are the main producers of ECM proteins in the heart and function as local immune modulators [105].

### Cell origins and types

The most common technique to identify cell source is the use of a fate map that represents the developmental history of each cell in the adult body [134]. Methods for fate mapping include labeling cells with dyes, creation of chimeric tissues from different species, detection of specific gene sequences (e.g., sex-mismatch transplantation), and the use of transgenic reporter systems (e.g., β-galactosidase) [47]. Cardiac fibroblasts have been tagged using transgenic mouse models, including the MHC-Cre skeletal muscle cell graft model that generated a subpopulation of skeletal-cardiac hybrid fibroblast cells, giving evidence for epithelial–mesenchymal transition origin [93, 135]. Even though the use of transgenic models to fate map cells is the current state of the art, it is limited since each model targets only one gene. A model where tagged cardiac fibroblasts from different sources could be visualized in real time would be ideal but is not yet feasible in adult mammals.

Fibroblasts are widely distributed within the body and display significant differences among organs and tissues. For example, fibroblasts from the atria respond differently to pathological stimuli compared with fibroblasts from the ventricle, denoting evidence of cellular diversity [7]. These differences result from the fact that cardiac fibroblasts can originate from different cellular sources depending on the stage of heart development and cellular context: homeostasis versus injury [33].

**Fig. 1:**
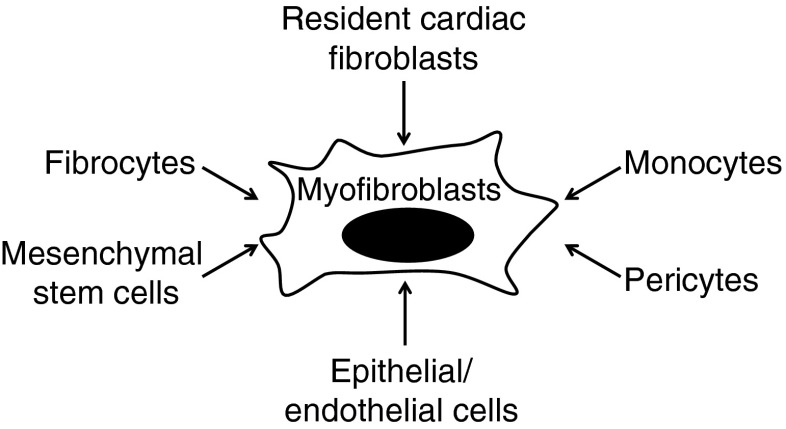
Origins of cardiac myofibroblasts. Cardiac myofibroblasts originate from a wide array of cell sources, including resident cardiac fibroblasts, bone marrow-derived fibrocytes, mesenchymal stem cells, epithelial/endothelial cells, pericytes, and monocytes

Following injury, myofibroblasts are induced by a variety of cell lineages and are the primary contributor of ECM. In addition to resident cardiac fibroblasts, myofibroblasts originate from bone marrow-derived fibrocytes, mesenchymal stem cells, epithelial/endothelial cells, pericytes, and monocytes (Fig. 1) [55]. Upon injury, resident cardiac fibroblasts are activated and differentiated into myofibroblasts by a series of inflammatory mediators and mechanical alterations [115]. Friendenstein and colleagues presented the first in vitro evidence for the bone marrow origin of fibroblasts about 40 years ago [31, 65]. They cultured bone marrow cells in media with fetal bovine serum and identified the resulting colonies as fibroblasts. Circulating cardiac fibroblast progenitor cells derived from the bone marrow have recently been termed fibrocytes [115]. Mesenchymal stem cells have pluripotent differentiation potential and are capable of differentiating into myofibroblasts [10]. Another origin of myofibroblasts is the epithelial/endothelial–mesenchymal transition (EMT), which describes tissue generation of myofibroblasts originating from organ epithelium (epithelial cells) and endothelium (endothelial cells) [54]. Pericytes play an important role in microvessel formation, maturation, and stability. In response to injury, pericytes can also become myofibroblasts [28]. Very recently, Trial and colleagues showed that monocytes infiltrating the heart can differentiate into fibroblasts [112]. This demonstration of conversion between mature cells opens an exciting new direction for LV remodeling research.

### Myofibroblast activation and differentiation

Myofibroblasts are essential for wound healing and have a contractile phenotype, sharing characteristics of smooth muscle cells [68]. This cell type is normally not present at high numbers in the healthy myocardium, but are a predominant cell type in the infarcted tissue after day 3 post-MI. Myofibroblasts express large amounts of α-smooth muscle actin (α-SMA), vimentin, desmin, and fibronectin extra domain A (EDA) markers [5]. The most commonly used molecular marker for myofibroblasts is α-SMA [41]. Expression of α-SMA increases myofibroblast contractile activity over 2-fold in vitro [39]. The mechanism behind this process is still unclear; however, the α-SMA specific N-terminal amino acid sequence AcEEED plays a central role in inducing cell contractibility. In vivo and in vitro studies show that delivery of the peptide AcEEED selectively inhibits α-SMA expression and reduces myofibroblast contractile activity [40]. Uncontrolled proliferation and/or activation of myofibroblasts results in tissue fibrosis. Therefore, identifying the mechanisms of action of the peptide AcEEED may help to understand the mechanisms involved in tissue remodeling, particularly in conditions of pathological fibrosis.

Transforming growth factor (TGF)-β is a major promoter of myofibroblast activation and differentiation. TGF-β induces the expression of α-SMA, modulates the expression of adhesive receptors, and enhances the synthesis of ECM molecules including collagen I and fibronectin EDA [43]. The pathway leading to TGF-β activation has been extensively studied. Proteases including plasmin, MMP-2, and MMP-9 cleave latent TGF-β to generate the active form [6]. Thrombospondin (TSP)-1 is also a key TGF-β activator, which acts by disrupting the noncovalent bond between the latency-associated peptide and the TGF-β molecule [71, 83]. Active TGF-β binds to TGF-β receptor type II and results in phosphorylation of receptor-activated Smads. Phosphorylated Smads (Smads 2, 3, and 4) form heteromeric complexes that translocate into the nucleus and result in efficient TGF-β signal transduction [86]. Activation of the Smad3 signaling pathway has been reported to stimulate ECM protein synthesis and TIMP upregulation in myofibroblasts [120].

The capability of TGF-β to induce myofibroblast differentiation is regulated by matricellular and ECM proteins, including tenascin-C, periostin, TSP-1, connective tissue growth factor (CTGF), secreted protein acidic and rich in cysteine (SPARC), and fibronectin. Tenascin-C, a matricellular protein that is upregulated post-MI, promotes recruitment of myofibroblasts in the early stages of myocardial repair through stimulation of cell migration and differentiation [110]. Periostin promotes myofibroblast recruitment and collagen synthesis, and periostin null mice have increased rates of LV rupture post-MI due to reduced collagen deposition [87, 104]. TSP-1 has been associated with myofibroblast maturation and fibrogenesis in a model of pressure-overloaded heart [128]. CTGF enhances TGF-β-induced fibroblast differentiation into myofibroblasts [19]. SPARC deficiency results in impaired fibroblast activation and decreased ECM production post-MI, thus facilitating LV rupture [79]. Serini and colleagues showed that fibronectin EDA deposition precedes α-SMA expression during granulation tissue evolution and during in vitro TGF-β fibroblast stimulation and differentiation, with the degree of myofibroblast differentiation proportional to the levels of fibronectin EDA [102]. Moreover, a fibronectin EDA blocking antibody inhibits TGF-β induced expression of α-SMA and collagen I in cultured cardiac fibroblasts, implying that fibronectin EDA mediates TGF-β-induced fibroblast differentiation [102]. From these studies, it is clear that matricellular proteins regulate several aspects of myofibroblast function.

### Myofibroblast functions

The myofibroblast has two main functions post-MI: provide mechanical strength to the scar by secreting new ECM proteins to replace the damaged myocardium and stimulating infarct contraction and produce factors that regulate the inflammatory and fibrotic responses. Myofibroblasts directly contact with ECM proteins through stress fibers at cell-integrin-matrix junction sites and with other cells via *N*-cadherin-type adherens junctions [38, 42]. These junctions increase myofibroblast contractility that is essential for structural integrity and support the new matrix to strengthen the infarct scar.

Post-MI, myofibroblasts migrate into the damaged tissue and synthesize new ECM. The activation stimuli come from pro-inflammatory cytokines and hormones, which are released by inflammatory and resident cells and from changes in the mechanical microenvironment [91, 111]. Following MI, cardiac fibroblasts respond to the loss of ECM integrity, increased mechanical stress, and increased levels of platelet-derived growth factor by adopting a partially differentiated phenotype known as the proto-myofibroblast [111]. Subsequently, myofibroblasts enhance their proliferative and migratory rates, secrete ECM proteins, synthesize MMPs, and regulate expression of cytokines and growth factors, such as tumor necrosis factor-α, interleukin (IL)-1, IL-6, and TGF-β [91].

In summary, myofibroblasts embody a unique, yet developmentally diverse, population of cells that play a major role in post-MI LV remodeling. Enhanced understanding of the cell source and the myofibroblast regulatory mechanisms can facilitate the generation of therapeutic targets for this multifunctional cell type to optimize infarct scar formation and prevent progression to heart failure.

## MMPs

MMPs are a group of proteolytic enzymes consisting of 25 known members. These proteins are generally released in their zymogen form, with the exception of MMP-11 and MMP-28 [46]. Structurally, the MMPs typically consist of four domains, which include a pro-domain, a catalytic domain, a hinge region, and a hemopexin domain [121]. MMP-2 and MMP-9 also have a fibronectin type II-like domain inserted into the catalytic domain that facilitates collagen cleavage [121]. With the exception of MMP-23, these enzymes are activated by a cysteine switch mechanism; disassociation of cystein-73 residue from zinc atom is required for exposure of the catalytic site, which is the switch that leads to the activation of the MMP [117, 121].

MMPs have been categorized into five groups based loosely on in vitro substrate specificity and localization: collagenases, gelatinases, stromelysins, matrilysins, and membrane type MMPs [89]. The role of MMPs in cardiac repair has been a focus of our group [46, 63]. In this review, we will focus on the role of MMP-1, MMP-2, MMP-3, MMP-7, MMP-8, MMP-9, MMP-13, MMP-14, and MMP-28, which are the MMPs that have been evaluated post-MI (Table 1).

**Table 1:** MMPs and TIMPs in the MI setting

MMP	Post-MI expression	Cellular source	Roles
1	↑	Myocyte, macrophage, and fibroblast	Predicts LV dysfunction and dilation in MI patients
2	↑	Myocyte, fibroblast, myofibroblast, vascular smooth muscle cells, and endothelial cell	↓ survival, ↑ LV rupture, and ↑ macrophage infiltration
3	↑	Myocyte, fibroblast, and macrophage	Correlates with LV dysfunction and mortality in MI patients, and activates MMP-1, MMP-3, MMP-7, MMP-8, MMP-9, and MMP-13
7	↑	Myocyte and macrophage	↓ survival, ↓ conduction velocity, and activates MMP-1, MMP-2, MMP-7, and MMP-9
8	↑	Neutrophil and macrophage	↑ LV rupture, ↑ neutrophil infiltration, and degrades collagen
9	↑	Myocyte, fibroblast, neutrophil, macrophage, vascular smooth muscle cell, and endothelial cell	↑ LV dilation, ↓/↑ LV function, ↑/↓ inflammation, and ↑ collagen deposition
13	↑	Fibroblast and macrophage	Activates MMP-9
14	↑	Myocyte, fibroblast, and myofibroblast	↓ LV function, ↓ survival, ↑ fibrobsis, and activates MMP-2 and MMP-13
28	↓ in myocyte ↑in macrophage	Myocyte and macrophage	↑ LV function, ↓ LV rupture, ↓ mortality, ↑ M2 macrophage polarization, and ↑ collagen deposition and cross-linking
TIMPs			
1	↑	Myocyte and fibroblast	↓ LV dilation
2	↑	Fibroblast	↓ LV dilation, ↑ LV function, ↓ infarct size, ↓ inflammation, and ↓ collagen disorganization
3	↓	Fibroblast	↓ LV dilation, ↑ LV function, ↓ LV rupture, ↓mortality, ↓ infarct size, ↑ myofibroblast number, ↑ collagen deposition, and ↓ MMP activity
4	↓	Myocyte	↓ LV rupture and ↓ mortality

*MMPs* matrix metalloproteinases, *TIMPs* tissue inhibitors of metalloproteinases, *MI* myocardial infarction, *LV* left ventricle

### MMP-1

MMP-1 (fibroblast collagenase or collagenase-1) was the first MMP discovered and named in 1966 [85]. MMP-1 has latent (57/52 kDa) and active (49/37 kDa) forms. MMP-1 can be expressed myocytes, macrophages, and fibroblasts [63]. Rodents do not express the same MMP-1 gene expressed by humans but rather have homologous forms MMP-1a and MMP-1b. MMP-1 cleaves collagen, gelatin, laminin, as well as non-ECM substrates such as complement C1q, IL-1β, and tumor necrosis factor-α [63, 121]. MMP-1 activity in the infarct area has been reported to increase 2 days after MI, peaking at day 7 [17]. This expression pattern indicates that MMP-1 may regulate the late inflammatory response and ECM turnover. In patients with acute MI, plasma MMP-1 levels are positively associated with LV dysfunction and dilation, implying that increased collagenolytic activity facilitates impairment of LV function [88].

### MMP-2

MMP-2 (gelatinase A or 72 kDa gelatinase) has a 72-kDa latent and a 66/62-kDa active form [46]. MMP-2 is expressed by myocytes, fibroblasts, myofibroblasts, vascular smooth muscle cells, and endothelial cells [63]. Substrates for MMP-2 include both extracellular and intracellar proteins, including aggrecan, citrate synthase, elastin, fibronectin, fusion protein, IL-1β, prolysyl oxidase, and other MMPs such as MMP-1, MMP-9, and MMP-13 [46]. Myosin light chain and troponin I are intracellular substrates for MMP-2 and highlight the interaction between some MMPs and cardiomyocytes [63, 98]. In animal studies, MMP-2 activity peaks by day 7 and declines to normal levels by day 14 after MI in the LV infarct area [63]. MMP-2 null mice subjected to MI have reduced macrophage infiltration in the infarct region, a lower rate of LV rupture, and improved survival in comparison to wild type mice [76]. This suggests a role for MMP-2 in regulating the entry of inflammatory cells in the infarcted area post-MI. Reduced ECM degradation in the absence of MMP-2 may explain the reduced rupture phenotype observed in MMP-2 null mice. In humans, post-MI plasma MMP-2 levels were surprisingly inversely correlated with LV volumes, and a larger cohort study is warranted before these results are interpreted [107].

### MMP-3

MMP-3 (stromelysin-1) has 59/57 kDa latent and 48 kDa active forms. MMP-3 has casein and proteoglycan degrading properties and is expressed by myocytes, fibroblasts, and macrophages [63, 121]. MMP-3 activates MMP-1, MMP-3, MMP-7, MMP-8, MMP-9, and MMP-13, indicating crosstalk within the family members [121]. MMP-3 increases within 3 days post-MI in rats and declines over the next 14 days [64, 95]. In acute MI patients, plasma MMP-3 levels are positively associated with patient age and sex (higher in male patients), creatinine levels, and hypertension, but negatively correlate with LV ejection fraction [51]. As a result, MMP-3 has been suggested to be an independent predictor of LV systolic dysfunction and mortality in MI patients [51].

### MMP-7

MMP-7 (matrilysin) is the smallest MMP, with 28 kDa latent and 19 kDa active forms. It is mainly expressed by the myocyte and macrophage. MMP-7 cleaves several ECM proteins and primarily activates MMP-1, MMP-2, MMP-8, and MMP-9 [121]. Post-MI, MMP-7 increases robustly in macrophages [121]. Our lab showed that MMP-7 deficiency improves mouse survival post-MI, but not by attenuating LV dilation [60, 63]. Interestingly, MMP-7 deficiency protected mice from reduced conduction velocity, and MMP-7 was shown to directly bind and process connexin-43. Using a proteomic strategy, we identified fibronectin and tenascin-C as additional in vivo MMP-7 substrates in the infarcted LV [15]. In addition, MMP-7 may indirectly regulate the expression of peroxiredoxin-1, peroxiredoxin-2, and peroxiredoxin-3 through nonenzymatic mechanisms [15].

### MMP-8

MMP-8 has 64 kDa latent and 58 kDa active forms. Although originally named neutrophil collagenase, MMP-8 is actually secreted by both neutrophils and macrophages [63]. MMP-8 cleaves fibrillar collagens by binding collagen type I α1 and α2 chains, thus promoting cell migration [59]. MMP-8 mRNA increases as early as 6 h. after MI. MMP-8 protein levels increase 2 weeks post-MI at both border and infarcted regions, and persist through 4 months, indicating its involvement in the early and late remodeling responses [90]. MMP-8 levels are higher in patients with LV rupture than those without rupture post-MI, indicating that MMP-8 may promote infarct rupture in humans by degrading collagen [116].

### MMP-9

MMP-9 (gelatinase B or 92 kDa gelatinase) is one of the first MMPs found to be expressed in infiltrating neutrophils and macrophages present in the infarct area [46, 70]. MMP-9 is also expressed by myocytes, fibroblasts, vascular smooth muscle cells, and endothelial cells- although at log-fold lower amounts compared with the infiltrating leukocytes [129]. In humans, MMP-9 has 92 kDa latent and 88 kDa active forms. In mice, MMP-9 has 105 kDa latent and 95 kDa active forms. MMP-9 is known to degrade a variety of proteins, including aggrecan, collagen, elastin, fibronectin, galectin-3, IL-1β, laminin, SPARC, as well as activate TGF-β1 by processing the latent TGF-β binding protein [46, 133]. An increase in MMP-9 levels correlates with the development of LV dysfunction [3]. Targeted deletion of the MMP-9 gene in mice leads to decreased collagen accumulation and attenuated LV dilation post-MI [24]. Of note, overexpression of MMP-9 only in macrophages also leads to beneficial effects post-MI (e.g. attenuated inflammatory response and improved LV function), suggesting various levels of regulation of post-MI healing process by MMP-9, depending on cell source and timing of expression [132].

### MMP-13

MMP-13 (collagenase-3) has 60 kDa latent and 48 kDa active forms. Fibroblasts and macrophages are reported to express MMP-13 [46, 63]. MMP-13 mRNA amounts remain stable following MI through 16 weeks in the rat, but MMP-13 activity increases 72 h. post-MI, indicating that MMP-13 activity can increase without affecting gene expression [46]. Not much is known about MMP-13 functions, mechanism of action, or how it affects LV remodeling post-MI; however, it is known to stimulate MMP-9 activation [46, 121].

### MMP-14

MMP-14 (membrane type 1 MMP; MT-1 MMP) has 65 kDa latent and 54/45/40 kDa active forms [63]. MMP-14 is expressed by cardiomyocytes, fibroblasts, and myofibroblasts. It cleaves collagen, fibronectin, gelatin, and can activate MMP-2 and MMP-13 [121]. MMP-14 increases 20-fold at 3 days post-MI and 50-fold at 16 days post-MI in non-infarcted LV regions, indicating critical roles in both early and late remodeling events [18, 61, 63]. Furthermore, in animal studies the increase in MMP-14 post-MI is associated with significant cardiac fibrosis, reduced LV function, and lower survival [63].

### MMP-28

MMP-28 (epilysin) is the newest identified member of the MMP family and was first cloned in 2001 [67]. The furin-like proprotein convertase processes the 58 kDa pro-form of MMP-28 to its 48 kDa active form, which is then secreted into the extracellular environment [69]. The 3 known substrates for MMP-28 are casein, Nogo-A (a myelin component), and neural cell adhesion molecule-1 [73, 121]. MMP-28 is highly expressed in the normal heart, suggesting roles in normal tissue homeostasis. However, MMP-28 null mice do not show an abnormal heart phenotype in the unstressed setting [69]. We have recently shown that MMP-28 derived from macrophages increases at 5 days post-MI, although total levels of the protein in the infarct decrease due to the loss of myocytes [69]. MMP-28 deletion worsens LV dysfunction and mortality as a result of increased cardiac rupture rate, which is strongly associated with impaired M2 macrophage activation and ECM deposition [69]. This study introduces the idea that not every MMP is detrimental and reinforces the concept that selective MMP inhibitors are warranted to inhibit individual MMPs.

## TIMPs

TIMPs are potent endogenous inhibitors of MMP activity and include four homologous members: TIMP-1, TIMP-2, TIMP-3, and TIMP-4. TIMPs play important roles in LV remodeling post-MI by directly inhibiting MMP activity and indirectly regulating remodeling processes (Table 1).

### TIMP-1

TIMP-1, a 29-kDa glycoprotein, is secreted by myocytes and fibroblasts. TIMP-1 mRNA increases in the infarct region within 6 h. following MI and declines after 2 days with no detectable alterations in regions remote from the infarct [17, 63]. Compared with wild-type counterparts, TIMP-1 knockout mice show larger LV volumes, which can be reversed by MMP inhibition [44]. This indicates that TIMP-1-dependent beneficial effects are mediated by inhibiting MMP activity.

### TIMP-2

TIMP-2 (28 kDa), which is primarily secreted by fibroblasts, is highly expressed in the normal myocardium. In addition to suppressing MMP activity, TIMP-2 is the only TIMP that is required for cell surface activation of pro-MMP2, suggesting both anti- and pro-activating roles [49]. TIMP-2 mRNA increases post-MI whereas the protein level does not undergo major change. Kandalam et al showed that the absence of TIMP-2 post-MI was associated with greater infarct expansion and LV dilation, worse LV dysfunction, and more severe inflammation and collagen disarray [49]. This could be mainly attributed to its inhibitory action on MMP-14. Therefore, TIMP-2 supplementation may provide a potential strategy in attenuating LV remodeling post-MI.

### TIMP-3

TIMP-3 (24 kDa) is ECM bound and has been shown to be a powerful inhibitor of all MMPs. TIMP-3 is highly expressed in the normal heart (secreted by fibroblasts). TIMP-3 protein levels decrease in both animals and patients with MI. Post-MI, TIMP-3 deletion has been demonstrated to increase mortality as a result of enhanced cardiac rupture, greater infarct expansion, exacerbated LV dilation, and worse LV dysfunction [48]. TIMP-3 null mice subjected to MI also showed reduced collagen synthesis, disorganized collagen deposition, decreased myofibroblast number in the infarct area, and increased MMP activity [37, 48]. MMP inhibition reverses the effects observed in the TIMP-3 null mice. Thus, proteolytic activity early post-MI is a promoting factor for subsequent adverse LV remodeling and dysfunction, and timing of intervention to improve the LV response to MI may be critical in producing a beneficial outcome [48].

### TIMP-4

In addition to having a 24-kDa form, TIMP-4 has a 28-kDa glycosylated form reported to be expressed by cardiomyocytes [63]. Unlike other TIMPs, TIMP-4 mRNA does not increase post-MI but protein levels reportedly decrease in weeks 1 and 4 post-MI [9, 118]. Similarly to other TIMPs, TIMP-4 deletion results in increased LV rupture and mortality, which can be prevented by MMP inhibition [53]. Plasma TIMP-4 levels, but not TIMP-1 or TIMP-2 plasma levels, has been suggested to predict LV remodeling in MI patients [124].

Taken together, studies clearly show the importance of MMPs and TIMPs post-MI and demonstrate that their protein and activity levels change during different phases of the wound healing process to regulate LV remodeling. Additional studies that explore the temporal and spatial patterns of MMP and TIMP expression will expand our understanding of the mechanisms of LV remodeling.

## Structural ECM

Fibrillar collagens, fibronectin, and laminins comprise the structural component of the ECM, which is important in maintaining architecture and preserving normal LV function [68]. Several functional domains within structural ECM or ECM-derived fragments serve critical roles in LV remodeling in the MI setting (Table 2) [68].

**Table 2:** Roles of ECM proteins increased post-MI

Name	Roles
Structural ECM	
Collagen I and III	Scar components and correlates with diastolic dysfunction and mortality risk
Collagen IV and V	Basement membrane components
Collagen VI	↓ LV function, ↑ apoptosis, and ↑ collagen deposition
Fibronectin EDA	↑ LV dilation, ↓ LV function, ↑ inflammation, ↑ MMP activity, and ↑ myofibroblast numbers
Laminin	Basement membrane component and negative correlation with LV function
Matricellular proteins	
CCN-1	↑ apoptosis, ↓ inflammation, and ↓ fibrosis in other models
CCN-2/CTGF	↓ infarct size in an ischemia/reperfusion model
CCN-4/WISP1	↓ myocyte apoptosis, ↑ hypertrophy, and ↑ fibroblast proliferation (in vitro)
Osteopontin	↓ LV remodeling, ↓ LV dilation, ↑ collagen deposition, and ↑ angiogenesis
Periostin	↑ LV function, ↓ LV rupture, ↓ fibrosis, and ↑ regeneration
SPARC	↑ LV function, ↓ LV rupture, ↓ mortality, ↑ macrophage infiltration, and ↑ scar organization
Tenascin-C	↑ LV remodeling, ↓ LV function, ↑ fibrosis, and ↑ fibroblast function
Thrombospondin-1	↓ LV remodeling, ↑ LV function, and ↓ inflammation
Proteins with matricellular functions	
Galectin-3	Positive correlation with infarct size, heart failure, and 30-day mortality
Biglycan	↓ LV dilation, ↑ LV function, ↓ LV rupture, ↓ mortality, ↑ LV tensile strength, and ↑ scar organization
Decorin	↓ LV dilation, ↑ LV function, ↓ infarct size, ↓ hypertrophy, and ↑ scar organization
Syndecan-1	↓ LV remodeling, ↑ LV function, ↓ inflammation, ↓ MMP activity, and ↑ scar quality
Syndecan-4	↑ LV function, ↓ LV rupture, ↓ mortality, ↑ inflammation, and ↑ ECM deposition
Vitronectin	Positive correlation with MI severity

*ECM* extracellular matrix, *MI* myocardial infarction, *LV* left ventricle, *CTGF* connective tissue growth factor, *SPARC* secreted protein acidic and rich in cysteine

### Collagens

Collagens are the most abundant ECM proteins in the LV; and of 28 subtypes, collagen type I is the most abundant [12]. Following MI, collagen types I, III, IV, V, and VI increase in the infarct region, covering approximately 30 and 60 % of the infarct area at days 7 and 21 post-MI, respectively [62, 103]. In particular, collagen types I and III are major components of the myocardium scar [103]. The cardiac fibroblast is the major source of collagens in the post-MI setting. Proper collagen accumulation and assembly is essential for preventing progressive LV dilation post-MI. Collagen proteolysis post-MI is regulated by multiple MMPs that have collagenolytic activity, including MMP-1, MMP-2, MMP-8, MMP-9, MMP-13, and MMP-14. The role of collagen peptides that are generated by MMP activity during LV remodeling, however, has not fully clarified.

Circulating collagen peptides are commonly used as cardiac biomarkers of myocardial fibrosis and as a prognosticator of cardiac function. Collagens are synthesized as procollagen forms, which are secreted into the interstitial space where they undergo cleavage of their end-terminal propeptide sequences to enable collagen fiber formation [123]. The collagen terminal propeptides (e.g., amino (*N*)-propeptide and carboxy (*C*)-propeptide) are released into the circulation by the action of specific procollagen *N*- and *C*-proteinases [122]. The most commonly used biomarker for quantification of collagen type I synthesis is its 100 kDa C-terminal propeptide, with serum levels positively associated with diastolic dysfunction [74]. The N-terminal propeptide of collagen type III is a 42-kDa peptide that is widely used as a marker for collagen type III synthesis [97]. Elevated serum levels of this peptide have been positively correlated with increased mortality risk and heart failure [16].

The non-fibrillar collagens, such as collagen types IV and V, organize the basement membrane structure. Collagen type IV levels increase in both infarcted and non-infarcted area post-MI [103]. Collagen type VI increases in the infarcted area within days post-MI, and then accumulates in both infarct and non-infarct area [66]. Surprisingly, absence of collagen type VI improves LV function, structure, and remodeling after MI by inhibiting apoptosis and collagen accumulation [66]. This indicates that collagens not only constitute the major components of the scar, but also regulate cardiac healing.

### Fibronectin

Fibronectin is a multifunctional adhesive glycoprotein produced by fibroblasts, macrophages, and endothelial cells in response to tissue injury. Fibronectin regulates cell shape and movement. For example, fibronectin guides macrophages into wound areas. Fibronectin contains an alternatively spliced exon encoding type III repeat EDA that is only expressed during development or after injury [68]. EDA can bind both toll-like receptor-2 and toll-like receptor-4 and activate monocytes in vitro [100]. EDA injection into murine joints activates nuclear factor-κB, resulting in enhancement of the inflammatory response [35]. EDA also regulates cell adhesion and proliferation and stimulates TGF-β pathway, which leads to generation of the myofibroblast phenotype [58, 102]. Interestingly, EDA-null mice with MI exhibit less LV dilation and better LV function compared with wild-type mice [2]. EDA-null mice also showed the reduced inflammation, MMP-2 and MMP-9 activities, and myofibroblast differentiation. These results suggest that EDA plays a critical role in adverse LV remodeling post-MI. Fibronectin also contains the extra domain B (EDB), which is expressed during embryogenesis [32]. Although the role of EDB in the MI setting has not been evaluated, EDB is highly expressed in normal tissue during angiogenesis, suggesting a role for EDB during LV remodeling following MI. Recently, using a proteomic approach, we showed that fibronectin is an in vivo substrate of both MMP-7 and MMP-9 [15, 133]. These findings expand our understanding of protective mechanisms when MMP-7 or MMP-9 is deleted in the MI setting.

### Laminins

Laminins are essential components of basement membranes and are composed of α, β, and γ chains. At least 15 laminin isoforms have been identified to date. Laminin is the first ECM to appear in the embryo. Laminin anchors cells to the ECM and can bind to multiple receptor proteins, such as collagen type IV, dystroglycan, entactin, heparin, heparin sulfate, integrins, and syndecans [108]. Laminin levels increase in the infarct area at day 3 post-MI and peak at days 7 to11 [82]. In the MI setting, laminin is detected throughout the infarct region, and this wide distribution suggests that the role of laminin post-MI exceeds the maintenance of basement membranes [82]. The expression pattern of laminin post-MI is similar to that of collagen type IV [82]. Serum concentrations of laminin and collagen type IV in acute MI patients are higher than in patients with stable or no coronary artery disease [22], and correlate with the severity of LV dysfunction [22]. This suggests that serum laminin and collagen type IV levels may be surrogate markers of ECM remodeling post-MI.

## Matricellular proteins

Matricellular proteins are a family of nonstructural matrix proteins that are capable of regulating a variety of biological functions by interacting with cell surface receptors, growth factors, proteases, and structural matrix [4]. Under normal conditions, matricellular proteins are generally present at very low expression levels. Following injury (e.g., MI), their expression increases substantially, implying a role in the repair response [68]. Matricellular proteins consist of CCN family members, osteopontin, periostin, SPARC, tenascins, and TSPs. Their roles in MI-induced LV remodeling have been extensively reviewed by several groups (summarized in Table 2) [29, 68]. Some proteins, such as galectins, small leucine-rich proteoglycans (SLRPs), syndecans, and vitronectin, share matricellular structure and/or functions, but have not been assigned to the family of matricellur proteins. In the following section, we will summarize current knowledge on their functions in MI-induced LV remodeling.

### Galectin-3

Galectins are composed of a family of evolutionarily conserved glycan-binding proteins and can bind to the carbohydrate portion of glycoproteins and glycolipids in the cell surface. Based on their structure and the number of carbohydrate recognition domain, galectins have been divided into three groups: (1) prototype galectins: galectin-1, galectin-2, galectin-5, galectin-7, galectin-10, galectin-11, galectin-13, and galectin-14; (2) tandem-repeat galectins: galectin-4, galectin-6, galectin-8, galectin-9, and galectin-12; and (3) chimera galectin-3 [131].

Among those, galectin-3 is the most widely investigated member associated with cardiovascular disease. Galectin-3 is a β-galactoside binding lectin primarily expressed by activated macrophages and serves as a novel marker for inflammation and fibrosis [25]. Weir and colleagues reported that plasma galectin-3 levels increased significantly following MI (mean, 46 h after MI) and was positively associated with remodeling parameters in patients with supra-median baseline LV ejection fraction (>49.2 %) but not when LV ejection fraction was ≤49.2 % [125].

In patients undergoing primary percutaneous coronary intervention for ST elevation MI, circulating galectin-3 levels were significantly higher than in healthy subjects, and elevated circulating level of galectin-3 was the strongest independent predictor of the combined 30-day major adverse clinical outcome (defined as advanced congestive heart failure or 30-day mortality) [114]. Elevated galectin-3 levels 4 months after acute MI are associated with larger infarct sizes, lower global myocardial function as well as with higher concentrations of NT-pro brain natriuretic peptide, highlighting the potential of galectin-3 as a biomarker of adverse remodeling after acute MI [78]. In patients with first MI treated with primary percutaneous coronary intervention, serum galectin-3 levels were an independent predictor for re-infarction [109]. These studies demonstrate the utility of galectin-3 in assessing late phases of LV remodeling after MI.

### Small leucine-rich proteoglycans

The SLRPs are a family of SLRPs that are divided into five classes based on homologies at both the genomic and protein levels [14]. SLRPs have a central domain termed tandem leucine-rich repeats, which can bind to innate immune receptors. SLRPs act as endogenous danger signals, known as damage-associated molecular patterns, which activate the innate immune system and regulate sterile inflammation-associated disease [30]. Of the ten family members, biglycan and decorin are well investigated regarding their roles in cardiovascular disease. Biglycan and decorin bind to collagen types I, II, III, and VI, and regulate collagen fibrillogenesis and fibril diameter [23]. The decorin binding site on collagen type I has been identified near the C terminus, very close to one of the major intermolecular cross-linking sites of collagen heterotrimers [50].

Post-MI, cardiac biglycan expression is significantly upregulated peaking at day 7, denoting a possible role in cardiac repair [127]. In animal studies, biglycan deficiency led to increased mortality rates after MI as a result of LV rupture. The biglycan null mice also exhibited aggravated LV dilation and worse LV function at 21 days post-MI [127]. Ex vivo measurements demonstrated that LV tensile strength was compromised, attributed to impaired collagen matrix organization [127]. Colocalization of biglycan with collagen type I in the infarct region implicates biglycan in regulation of collagen deposition and appropriate cross-linking [130]. Therefore, biglycan is required for stable collagen assembly and scar formation to preserve LV function. Compared with wild-type, biglycan null cardiac fibroblasts exhibited enhanced proliferation and increased TGF-β receptor II expression and Smad2 phosphorylation. Biglycan deficiency increased fibroblast transformation to myofibroblast, characterized by increased incorporation of α-SMA into stress fibers, formation of focal adhesions, and contraction of collagen gels [80]. Neutralizing TGF-β activity reversed the pro-proliferative and myofibroblastic phenotype in the absence of biglycan [80]. This suggests that biglycan regulation of fibroblast phenotype and function is dependent on TGF-β signaling.

After MI, decorin deletion resulted in a wider distribution of collagen fibril sizes, resulting in a less organized and looser packed scar [126]. As such, decorin null mice exhibited a significant increase in scar size and right ventricular hypertrophy, as well as aggravated LV dilation and dysfunction [126]. These data demonstrate that decorin is required for proper collagen assembly, and that its absence leads to disorganized scar formation.

### Syndecans

Syndecans are highly conserved transmembrane heparan sulfate proteoglycans and include four family members: syndecan-1 (syndecan), syndecan-2 (fibroglycan), syndecan-3 (*N*-syndecan), and syndecan-4 (ryudocan and amphiglycan). Structurally, syndecans are composed of an extracellular ectodomain, a transmembrane domain, and a short cytoplasmic domain. The ectodomain can be shed from cells and become a soluble form, which is the reason why syndecans have been occasionally considered matricellular proteins [29, 96]. Syndecans bind to a wide range of ECM proteins, growth factors, chemokines, and anticoagulant proteins by heparan sulfate glycosaminoglycan chains, playing critical roles in regulation of tissue injury and repair [11].

Following MI in experimental animals, syndecan-1 expression in the LV is upregulated at 24 h, peaks at 7 days and declines thereafter [119]. Syndecan-1 deletion leads to adverse LV remodeling and dysfunction, accompanied by increased inflammation and activity of MMP-2 and MMP-9, decreased tissue transglutaminase activity, as well as increased collagen fragments and disorganization in mice subjected to MI [119]. In concordance, adenoviral gene expression of syndecan-1 rescued the effects observed in the absence of syndecan-1. These data suggest that increased syndecan-1 after MI protects from excessive inflammation and adverse infarct remodeling. A recent study showed that increased serum concentration of soluble syndecan-1 after MI was due to its increased expression in the infarcted myocardium [56].

Elevated plasma syndecan-4 levels in MI patients and the existence of syndecan-4 in the infarct region attracted research interest in investigating its role in MI-induced LV remodeling [52, 57]. Syndecan-4 null mice do not show morphological and functional cardiac phenotype changes under normal conditions [75]. However, syndecan-4 deletion results in worse LV function and increased cardiac rupture and mortality. The potential mechanisms are associated with reduced inflammation, impaired granulation tissue formation, and decreased ECM deposition in the infarct region [75]. In vitro, syndecan-4 deficiency results in inhibition of fibronectin-induced, but not TGF-β1-induced, fibroblast differentiation into myofibroblasts. The shed form of syndecan-4 acts as a dominant-negative inhibitor of endogenous syndecan-4 signaling. Accordingly, overexpression of the syndecan-4 shed form mimics the effects observed when syndecan-4 is deleted [75]. In summary, both syndecan-1 and syndecan-4 exert protective roles in the MI setting by containing the inflammatory reaction and modulating ECM deposition.

### Vitronectin

Vitronectin is an adhesive glycoprotein with multiple functions including blood coagulation, complement activation, binding to proteoglycans, and modifying the ECM [92]. In addition, vitronectin regulates cell differentiation, proliferation, migration, and morphogenesis [136]. Vitronectin can bind to a number of ligands/receptors, such as the urokinase receptor, integrin type cell adhesion receptors, growth factors, proteoglycans, plasminogen activator inhibitor, and high molecular weight kininogen [72, 92, 136]. The urokinase receptor regulates monocyte adhesion by direct binding to vitronectin, indicating a key role of vitronectin in regulating inflammation [77]. Plasma vitronectin concentration is elevated in patients with coronary artery diseases, showing a positive correlation with the extent of disease [26]. Another clinical trial showed that a higher percentage of patients with baseline vitronectin of ≥49.7 μg/mL had major adverse cardiovascular events (e.g., death, MI, or urgent revascularization) than patients with vitronectin of <49.7 μg/mL at 30 days [21]. Moreover, when baseline variables not predictive of major adverse cardiovascular event (e.g., troponin positive, history of congestive heart failure, diabetes, history of hypertension, and smoking status) were excluded from the multivariate model, only baseline vitronectin of ≥49.7 μg/mL and history of MI remained [21]. This indicates that vitronectin may serve as an independent predictor of adverse cardiovascular outcomes following acute stenting.

## Matricryptins

Matricryptins, biologically active fragments of the ECM, are generated from various mechanisms including enzymatic degradation, protein multimerization, absorption, cell-mediated mechanical forces, and denaturation [68]. Matricyrptins can be generated from any protein found in the extracellular space. Matricryptins regulate cell migration, proliferation, differentiation, morphogenesis, survival, ECM assembly, angiogenesis, tissue repair, and the inflammatory reaction [68, 94]. For example, neutrophils exert chemotaxis to fragments of collagen type IV [101]. Conversely, a matricryptin peptide from the α3 chain of collagen IV suppresses neutrophil activation [81]. MMP-9 cleaves the α3 chain of collagen type IV to produce tumstatin, which inhibits angiogenesis [36]. In addition, MMPs are reported to cleave collagen IV, generating both anti- and pro-angiogenic fragments [13]. These reports suggest that collagen peptides have key biological activity in regulating post-MI LV remodeling. In the post-MI setting, endostatin, a matricriptin generated from collagen type XVIII, is elevated, and neutralization of endostatin exacerbates LV remodeling and dysfunction [45].

Fibronectin fragments stimulate monocyte migration and cause a positive feedback loop by increasing MMP production [99]. In addition, macrophages activated with fibronectin fragments enhance the survival of injured cardiac myocytes [113]. Degradation of laminin by neutrophil elastase generates fragments that are chemotactic for neutrophils, thus establishing a positive feedback loop for neutrophil recruitment [84]. Laminin α1 fragment stimulates macrophage expression of MMP-9, and laminin α5 peptide is reported to induce a wide range of cytokines in macrophages leading to a chemotactic response [1, 27]. These data indicate that laminin and its fragments may play important roles in the inflammatory response post-MI. Research has just scratched the surface of matricryptin roles in the post-MI LV.

## Future directions

**Fig. 2:**
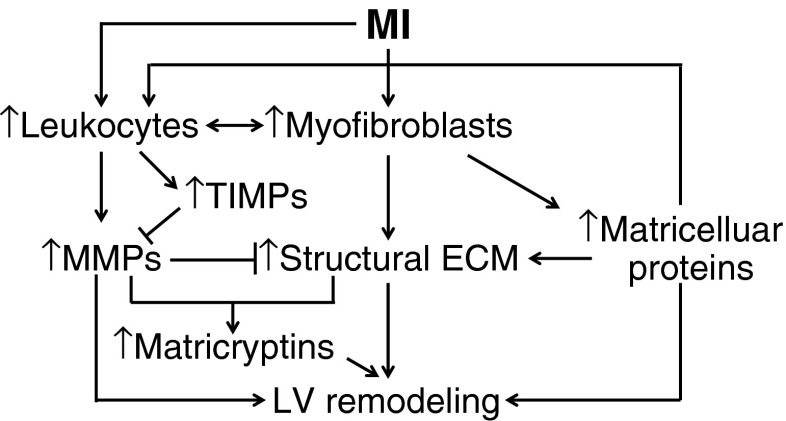
The mechanisms of ECM regulation of LV remodeling post-MI. Following MI, increased myofibroblasts secrete abundant ECM proteins. Structural ECM mainly constitutes the reparative scar. MMPs primarily derived from leukocytes degrade ECM constituents and suppress ECM synthesis, which, in turn, is inhibited by TIMPs. Nonstructural matricellular proteins modulate leukocyte infiltration and function and affect structural ECM assembly. In addition, matricryptins, biologically active fragments of ECM, are formed by MMP degradation of ECM to influence several aspects of LV remodeling

Myofibroblasts, as the major source of ECM, are indispensable to appropriate cardiac repair post-MI. Controlling myofibroblast activation and function may stimulate proper wound healing response, thus attenuating adverse or excessive LV remodeling and preserving LV function. Balanced structural ECM deposition and breakdown limits infarct expansion, LV aneurysm and rupture, and LV wall stiffness. This process is tightly regulated by MMP activity, which in turn is controlled by TIMPs. Nonstructural matricellular proteins modulate inflammation, angiogenesis, and scar formation by direct binding to cell surface receptors and matrix. The sophisticated ECM networks crosstalk with each other, with inflammatory cells and mediators, to coordinate cardiac repair process (Fig. 2) [29, 68].

Despite advances over the past two decades in understanding the mechanisms of LV remodeling post-MI, additional studies are still needed to further clarify several important aspects (Table 3). First, mapping the phenotypes of fibroblasts isolated from infarct hearts at different time points will provide clues to their roles in coordinating the ECM response. Second, the temporal and spatial expression, cellular source, and function of many MMPs after MI have not yet been studied. For example, of the 25 identified MMPs, the role of 16 MMPs in the MI setting is presently unknown. Third, a more inclusive list of MMP substrates may provide additional insights, particularly for the non-ECM molecules (e.g., cytokines and chemokines), activated or deactivated by MMPs [46]. This broadens the field of MMP activity that directly links with the inflammatory response. Proteomics strategies that target inflammatory mediators and ECM can provide a more thorough and high throughput identification of novel ECM and non-ECM substrates [20]. Moreover, the biological functions of the fragments derived from substrate cleavage need to be characterized. Finally, more information on the individual ECM constituents and their roles in the remodeling response is needed. There are likely hundreds of different ECM proteins in the post-MI LV. The individual roles as well as their functions as part of the entire remodeling response remain to be examined [68]. While in vitro experiments study the roles of individual component, additional knowledge regarding the interaction of various components in regulating remodeling in the context of the entire post-MI myocardium is needed. This will allow us to design inhibitors or agonists that have the best translational potential.

**Table 3:** Areas of research for future directions

1. Fibroblast and myofibroblast post-MI phenotypes, as spatial and temporal variations in phenotypes may explain functional differences in LV remodeling responses
2. Expression patterns and roles of the multitude of MMPs not yet studied
3. Identification and characterization of fragments derived from ECM and non-ECM substrates
4. Expression patterns and roles of ECM constituents

*MI* myocardial infarction, *LV* left ventricle, *MMPs* matrix metalloproteinases, *ECM* extracellular matrix

## Conclusions

In conclusion, MI remains a major cause of congestive heart failure. The extent of MI-induced LV remodeling is greatly dependent on the ECM regulation. Elucidating how the ECM regulates this transition of MI to heart failure will identify novel intervention targets that may help to stimulate an optimal wound healing response.
